# Induction of Endoplasmic Reticulum Stress by Prodigiosin in Yeast *Saccharomyces cerevisiae*

**DOI:** 10.3390/cimb46030116

**Published:** 2024-02-26

**Authors:** Sy Le Thanh Nguyen, Thi Hien Trang Nguyen, Thi Tuyen Do, Thi Thao Nguyen, Thanh Hoang Le, Thi Anh Tuyet Nguyen, Yukio Kimata

**Affiliations:** 1 Institute of Biotechnology, Vietnam Academy of Science and Technology, 18 Hoang Quoc Viet Road, Caugiay District, Hanoi 10600, Vietnam; nthientrang@ibt.ac.vn (T.H.T.N.); dttuyen@ibt.ac.vn (T.T.D.); ntthao@ibt.ac.vn (T.T.N.); hoanglt@ibt.ac.vn (T.H.L.); ntanhtuyet@ibt.ac.vn (T.A.T.N.); 2 Graduate School of Science and Technology, Nara Institute of Science and Technology, 8916-5 Takayama, Ikoma, Nara 630-0192, Japan

**Keywords:** prodigiosin, bacterial secondary metabolite, endoplasmic reticulum stress, unfolded-protein response, ROS, yeast, *Saccharomyces cerevisiae*

## Abstract

Prodigiosin, a red pigment produced by numerous bacterial species, exerts various antibiotic effects on prokaryotic and eukaryotic organisms. For instance, human carcinoma cell lines appear to suffer from endoplasmic reticulum (ER) stress in the presence of prodigiosin. Here, we demonstrated that prodigiosin also triggers the unfolded-protein response (UPR), which is a cytoprotective response against ER stress, in yeast *Saccharomyces cerevisiae*. An *S. cerevisiae* mutant carrying a UPR-deficient mutation was hypersensitive to prodigiosin. Our observations cumulatively indicate that protein folding in the ER is impaired by prodigiosin, illustrating a new mode of action.

## 1. Introduction

Prodigiosin is a secondary metabolite and red tripyrrole pigment produced by various bacteria such as the *Serratia*, *Vibrio*, *Hahella*, *Pseudoalteromonas*, and *Streptomyces* species (Figure S1) [1,2,3]. Prodigiosin has received considerable attention due to its diverse biological activities, which are related to its antibacterial, antifungal, antimalarial, immunosuppressant, and anticancer properties [2,3,4,5]. Therefore, it and its derivatives are promising candidates for use as therapeutic drugs.

Prodigiosin acts as an apoptotic agent in carcinoma cells [6,7]. As a possible antitumor mechanism, it acts as a proton-sequestering agent to destroy the intracellular pH gradient [8]. Moreover, prodigiosin has been proposed to affect various intracellular signaling pathways in mammalian cells. Its clinical potential for treating breast cancer may lie in its ability to inhibit Wnt/β-catenin signaling [9,10]. In addition, prodigiosin is reported to have the capacity to modulate the functions of p53 and p73 [11]. More recently, prodigiosin has been shown to inhibit intracellular transport and the recycling of cell surface receptors [12]. Another intriguing topic is that of prodigiosin provoking the endoplasmic reticulum (ER) stress response, which is also known to induce apoptosis, in carcinoma cells [13,14].

The ER is a membrane-surrounded cellular compartment in which secretory and transmembrane proteins are folded. Dysfunction of the ER, namely ER stress, is frequently accompanied by the ER accumulation of unfolded proteins and induces the ER stress response, which is also called the unfolded-protein response (UPR) [15]. The UPR is an intracellular signaling pathway that leads to the transcriptional induction of a number of genes, including those encoding factors for protein folding and modification in the ER. Although the UPR is widely believed to function in cytoprotection against ER stress, it often triggers apoptosis and paraptosis in mammalian cells [16]. Presumably, a merit of this phenomenon is the elimination of heavily stressed and damaged cells from the mammalian body.

While the UPR is commonly observed in eukaryotic cells, its mechanism was initially explored in detail through frontier studies using the yeast *Saccharomyces cerevisiae* as a model organism [17]. Ire1 is an ER-located transmembrane endoribonuclease that is conserved in eukaryotic species, and acts as an ER stress sensor to provoke the UPR. Unlike mammalian cells, *S. cerevisiae* does not have other ER stress sensors, such as activating transcription factor 6 (ATF6) and protein kinase R-like endoplasmic reticulum kinase (PERK) [18]. In ER-stressed cells, Ire1 detects ER-accumulated unfolded proteins and exerts strong endoribonuclease activity [18]. To the best of our knowledge, the sole target of Ire1 in *S. cerevisiae* is *HAC1* mRNA. When activated, Ire1 promotes the splicing of *HAC1* mRNA, which is then translated into a transcription factor that induces the expression of a number of UPR-target genes, including those carrying the UPR element (UPRE) on their promoter regions [17].

As for the relevance of prodigiosin to ER stress or the UPR, there are some unanswered questions. How does prodigiosin induce ER stress? Does prodigiosin provoke the UPR in eukaryotic species other than mammals? In order to answer these questions, here, we used *S. cerevisiae* as a model organism and demonstrated that prodigiosin inhibits protein folding in the ER, leading to UPR evocation. Thus, this study illustrates a new mode of action of prodigiosin, a prominent bacterial secondary metabolite with various antibiotic activities.

## 2. Materials and Methods

### 2.1. Chemical Materials

Prodigiosin was produced by *Serratia marcescens* M10 (QBN VTCC 910027) and purified basically as described previously [19] using a dual-step silica-gel column chromatography protocol (first-step solvent of toluene/ethyl acetate = 9:1 (*v*/*v*) and second-step solvent of toluene/ethyl acetate = 7:3 (*v*/*v*)). The stock solution of prodigiosin (0.1 mg/mL dimethyl sulfoxide (DMSO)) was stored at −20 °C under light-shielded conditions. Dithiothreitol (DTT) was purchased from Merck KGaA (Darmstadt, Germany).

### 2.2. Yeast Strains and Plasmids

In this study, we used the *S. cerevisiae* strain KMY1015 (*MATα*, *ura3*, *his3*, *leu2*, *trp1*, *lys2*, *ire1::TRP1*; Ref. [20]) or its derivative, KMY1516 (*MATα*, *ura3*, *his3*, *leu2::UPRE-GFP*, *trp1*, *lys2::UPRE-lacZ*, *ire1::TRP1*; Ref. [21]) carrying the *IRE1*-gene centromeric plasmid pRS313-IRE1 [21] as the *IRE1+* strains. The *ire1*-kockout genotype of these strains was confirmed by PCR before the experiments in this study. Other genotypes were confirmed based on auxotrophic requirements. For the *ire1∆* strains, we introduced the empty vector pRS313 [22] instead of pRS313-IRE1 into KMY1015 or KMY1516. The ∆III mutation of *IRE1* was introduced into pRS313-IRE1 using the in vivo gap repair technique [21]. For the UPRE-lacZ reporter assay, KMY1015 was transformed with the UPRE-lacZ reporter plasmid pCZY1 [23] before the introduction of the *IRE1* plasmids. To visualize the cellular localization of Kar2, an improved green fluorescent protein (GFP) sequence was inserted in-frame into the chromosomal *KAR2* gene, as described in Ref. [24]. For the *GAL1* promoter-driven expression of the ∆pro mutant version of *Rhizopus niveus* aspartic proteinase (RNAP-∆pro) [25], the plasmid pYPR3841U [26] was modified to carry the *LEU2*-selectable marker instead of *URA3* and introduced into *S. cerevisiae* strains.

### 2.3. Yeast Culturing

*S. cerevisiae* cells were cultured in synthetic dextrose (SD) medium containing 2% dextrose, 0.66% yeast nitrogen base w/o amino acids (Difco), and auxotrophic supplements. Cells were aerobically shaken at 30 °C in liquid SD medium, and stressor chemicals were added to the exponentially growing cultures, which were further incubated under the same conditions. For the expression of RNAP-∆pro under the control of the *GAL1* promoter, cells were cultured in synthetic galactose medium for 14 h.

### 2.4. UPR Assays

For the UPRE-lcaZ reporter assay, cellular β-galactosidase activity was measured, as previously described [23].

To check the splicing of *HAC1* mRNA, total RNA samples were extracted from yeast cells using the hot phenol method and subjected to reverse transcription using a poly(dT) oligonucleotide primer, as described previously [27]. The resulting cDNA samples were subjected to real-time polymerase chain reaction (PCR) using the intercalator method with two different *HAC1* primer sets [27]. One was used for amplification of the total *HAC1* species (5′-GCGTCGGACCAAGAGACTT-3′ and 5′-TCGTCGACTCTGGTACATTTTC-3′), and the other was used for amplification of the spliced version (5′-ACCTGCCGTAGACAACAACA-3′ and 5′-ACCTGACTGCGCTTCTGGAT-3′). The *HAC1* mRNA-splicing efficiency (%) was calculated using the following formula:

100 × [Abundance of spliced *HAC1* mRNA]/[Abundance of total *HAC1* mRNA]

Alternatively, the cDNA samples were subjected to PCR using the primer set to amplify the total *HAC1* species (5′-TACAGGGATTTCCAGAGCACG-3′ and 5′-TGAAGTGATGAAGAAATCATTCAATTC-3′), as described previously [27]. The PCR products were separated by electrophoresis on 2% agarose containing 0.0001% ethidium bromide. The DNA bands in the agarose gels were observed using the E-box gel documentation imaging system (Vilber, Collégien, France).

### 2.5. Inhibition Circle Assay

After spreading yeast cultures saturated overnight (100 µL culture for a 120 mm diameter plate) on the surface of SD agar plates, we punched the agar to make holes (approximately 8 mm in diameter), into which 100 µL of test chemical solutions were applied. After incubation at 30 °C for three days, the SD agar plates were measured to determine the diameter of inhibition zones using an electric caliper.

### 2.6. Fluorescence Microscopy

For GFP fluorescence, cells were observed under a Keyence BZ-9000E fluorescence microscope (Osaka, Japan) with a CFI Plan Apo λ100xH objective lens (Nikon, Tokyo, Japan).

### 2.7. Statistics

Values are presented as the mean and standard deviation from multiple biological replicates. To obtain *p* values, we performed a two-tailed unpaired *t*-test using Excel 2021 (Microsoft, Redmond, WA, USA).

## 3. Results

At the beginning of this study, we extracted and purified prodigiosin from an *S. marcescens* strain. The integrity and purity of the prodigiosin sample are shown in Figure S2. On thin-layer chromatography (TLC), the prodigiosin sample exhibited a single spot, the migration of which did not differ from that of a commercially purchased standard. In addition, the prodigiosin sample exhibited a single peak on high-performance liquid chromatography (HPLC). Based on the HPLC data, we estimated that the purity of the prodigiosin sample exceeded 98%. The ^1^H nuclear magnetic resonance (NMR) spectrum also identified the sample as prodigiosin. It should also be noted that aberrant peaks were not observed in the ^1^H NMR spectrum, confirming the purity of the prodigiosin sample.

**Figure 1:**
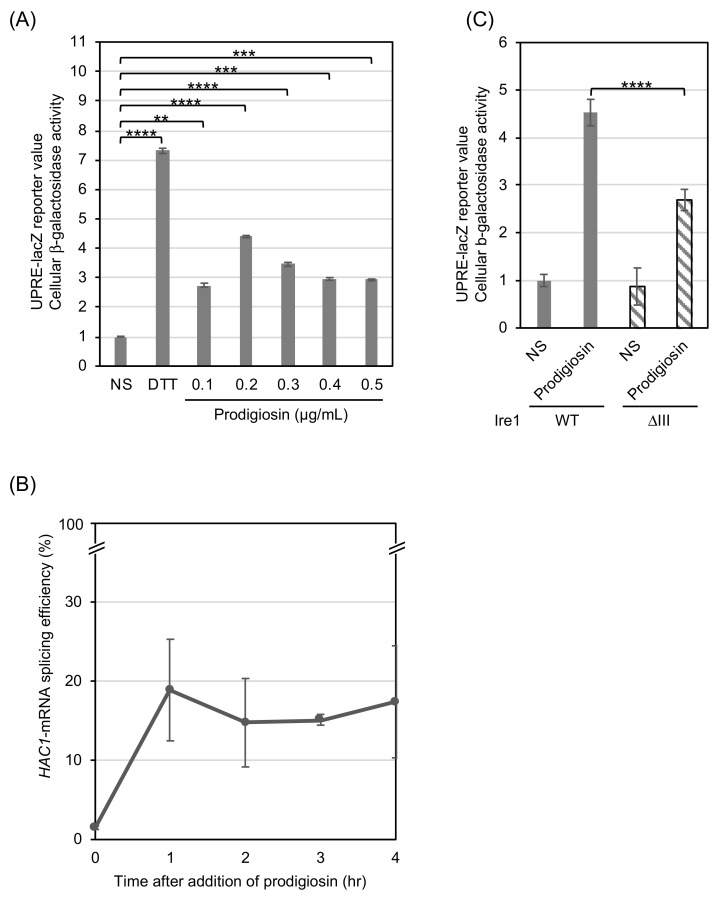
Prodigiosin activates the Ire1/*HAC1*-dependent UPR signaling pathway in *S. cerevisiae.* (**A**) Before measurement of cellular β-galactosidase activity, *IRE1+* cells carrying the UPR-lacZ reporter plasmid were treated with DTT (10 mM, 0.5 h) or prodigiosin (3 h), or remained unstressed (non-stress: NS). The resulting values were normalized against that of unstressed cells, with normality set at 1.0. (**B**) *IRE1+* cells were cultured in the presence of 0.2 µg/mL prodigiosin for the indicated periods, and total RNA samples were examined for *HAC1* mRNA splicing. The *HAC1* mRNA splicing efficiency indicates the relative abundance of spliced *HAC1* mRNA relative to that of total *HAC1* mRNA, which was calculated as described in the Materials and Methods. (**C**) Using *IRE1*+ cells and their ∆III mutant, a similar analysis to that in panel A was performed (NS or prodigiosin treatment (0.2 µg/mL, 3 h)). The resulting values were normalized against that of unstressed *IRE1*+ cells, with normality set at 1.0. **: *p* < 0.01, ***: *p* < 0.001, ****: *p* < 0.0001. In all panels, the averages and standard deviations were calculated from the data obtained from three biological replicates.

In the experiment shown in Figure 1A, we used *S. cerevisiae IRE1*+ cells carrying the UPRE-lacZ reporter plasmid, from which bacterial β-galactosidase was expressed under the control of UPRE. As expected, cellular β-galactosidase activity increased when DTT, which cleaves protein disulfide bonds and acts as a strong ER stressor, was added to the culture medium. Importantly, prodigiosin also induced the expression of the UPRE-lacZ reporter, suggesting ER stress evocation by prodigiosin. The UPRE-lacZ values seemed to be almost equally elevated by different concentrations of prodigiosin (from 0.1 to 1.0 mg/mL). Because the UPRE-lacZ value was highest, albeit not drastically, when prodigiosin was added into cultures at a concentration of 0.2 µg/mL, we employed this concentration of prodigiosin for the following experiments.

Next, we confirmed UPR evocation by prodigiosin in *S. cerevisiae* cells by monitoring *HAC1* mRNA splicing. When prodigiosin was added to the culture at a concentration of 0.2 µg/mL, *HAC1* mRNA splicing was induced within 1 h and continued for at least 4 h (Figure 1B and Figure S3).

Ire1 is known to directly sense ER-accumulated unfolded proteins for its activation upon ER stress. We previously reported that a partial deletion mutation in the luminal domain of Ire1, namely the ∆III mutation (deletion of a.a. 253-272), impairs the ability of Ire1 to detect unfolded proteins [28]. Figure 1C shows that the ∆III mutation compromised the prodigiosin-induced UPR. This observation suggests that prodigiosin provokes the UPR by disturbing protein integrity in the ER.

**Figure 2:**
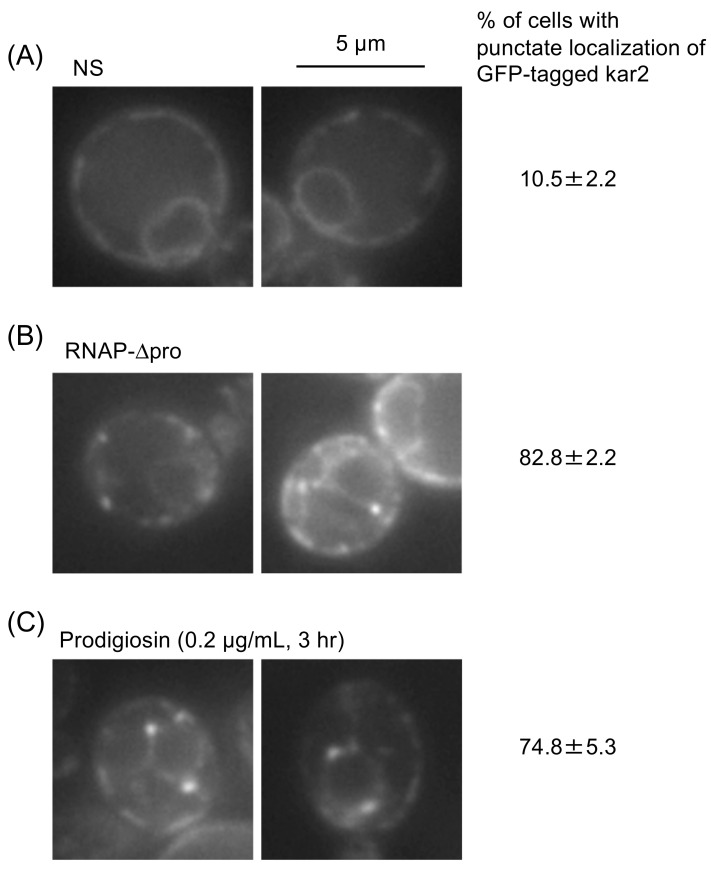
Prodigiosin causes aggregation of Kar2 in *S. cerevisiae* cells. *IRE1+* cells expressing GFP-tagged Kar2 were observed under a fluorescence microscope, as described in the Materials and Methods. Moreover, the number of cells showing a dot-like distribution of GFP-tagged Kar2 (such as the cells shown in panels **B** and **C**) was counted and divided by the total number of cells showing the fluorescent signal of GFP-tagged Kar2. At least 100 cells were counted, and data are presented as the average and standard deviation from three biological replicates. (**A**) Cells were cultured under non-stress (NS) conditions. (**B**) Cells containing the plasmid for the expression of RNAP-∆pro under the *GAL1* promoter were cultured in synthetic galactose medium for 14 h. (**C**) Cells were cultured in the presence of 0.2 µg/mL prodigiosin for 3 h.

Binding immunoglobulin protein (BiP) is a prominent molecular chaperone located in the ER. *S. cerevisiae* BiP is called Kar2 and is known to bind to unfolded proteins accumulated in the ER [26]. Unfolded proteins are sometimes aggregated, recruiting Kar2, which then exhibits punctate distribution [25,29,30]. In the experiment shown in Figure 2, we used *S. cerevisiae* cells that express Kar2 carrying the GFP tag just before the C-terminal HDEL ER-retention signal [24]. As previously reported [24], GFP-tagged Kar2 exhibited a typical ER-like double-ring localization pattern, indicating its diffusive distribution through the ER (Figure 2A). In contrast, GFP-tagged Kar2 partly exhibited dot-like localization in cells expressing RNAP-∆pro, which is a heterologous aberrant protein that accumulates and aggregates in the ER of *S. cerevisiae* cells [25]. Notably, a similar localization pattern was observed when cells were treated with 0.2 µg/mL prodigiosin for 3 h (Figure 2C).

Since the UPR is a cellular response to cope with ER stress, cells show hypersensitivity to ER stressors when carrying mutations that halt the UPR signaling pathway. In the experiment presented in Table 1 and Figure S4, we performed an inhibition circle assay to determine the sensitivity of *IRE1*+ and *ire1∆* cells to prodigiosin. As expected, prodigiosin formed larger growth inhibition zones on *ire1∆* cells than on *IRE1+* cells.

## 4. Discussion

As described in the Introduction, prodigiosin exhibits antibiotic activities against a wide variety of prokaryotic and eukaryotic species, indicating its potential for biomedical applications. Although prodigiosin is reported to have antifungal activity, to the best of our knowledge, its mode of action in fungal and yeast cells has been poorly documented. Meanwhile, it is also uncertain how prodigiosin induces ER stress, which finally leads to apoptosis, in mammalian carcinoma cells. Therefore, in the present study, we addressed the biological consequences of prodigiosin in *S. cerevisiae*, a widely used model organism, from the viewpoint of ER stress and UPR.

As shown in Figure 1, we demonstrated that prodigiosin provokes UPR, which was monitored by UPRE-lacZ reporter and *HAC1* mRNA-splicing analyses, in *S. cerevisiae* cells. Prodigiosin is likely to damage cells by inducing ER stress at least partly, because *ire1∆* cells were more sensitive to prodigiosin than *IRE1+* cells (Table 1).

It is widely accepted that ER stress causes the ER accumulation of unfolded proteins, which are directly detected by Ire1 to trigger the UPR [18]. Nevertheless, some ER stress stimuli, such as those causing membrane lipid-related abnormalities, are likely to induce UPR without yielding unfolded proteins in the ER [28,31]. The UPR, when mediated and not mediated by the ER accumulation of unfolded proteins, can be distinguished, as the former is compromised by the ∆III mutation of Ire1, which impairs Ire1’s ability to sense unfolded proteins [28]. We deduce that prodigiosin triggers UPR by yielding unfolded proteins in the ER, because the prodigiosin-induced UPR was compromised by the ∆III mutation. This insight has been supported by another observation indicating that prodigiosin caused punctate localization of Kar2, which is a hallmark of the ER accumulation of unfolded proteins, in *S. cerevisiae* cells (Figure 2) [25,29,30].

How does prodigiosin impair protein integrity in the ER? Considering its various antibiotic effects in both prokaryotic and eukaryotic cells [1,2,3,4,5], prodigiosin does not appear to be a sophisticated and specific inhibitor that blocks a selected biological process. Ref. [32] proposed that prodigiosin may be a hydrophobic agent that damages biological membranes. Moreover, Ref. [33] proposed that prodigiosin interacts abiotically with DNA. We presume that prodigiosin, which is composed of three pyrrole rings and a linear hydrocarbon chain (Figure S1), has chaotropic characteristics for interactions with a wide variety of biological molecules, possibly leading to protein denaturation.

The UPR induced by prodigiosin appeared to be weaker than that induced by DTT, and was not elevated even when the dose was increased (Figure 1A). This contrasts with the case of DTT, which strongly induces UPR in a dose-dependent manner [34]. We speculate that this difference is due to a difference in the modes of action of prodigiosin and DTT. DTT is believed to non-specifically cleave cysteine disulfide bonds, leading to the denaturation of a large portion of proteins that traverse the ER. In contrast, prodigiosin may impair the integrity of selected proteins. In agreement with this idea, the expression of a single aberrant ER-client protein induces the UPR only modestly, even when it is expressed from a strong gene expression promoter [35]. Nevertheless, to fully support this idea, it is necessary to determine the primary targets of prodigiosin in the *S. cerevisiae* ER, which is a future research question.

Another important question is whether ER stress is the major cause of prodigiosin toxicity in *S. cerevisiae*. In this study, we demonstrated that the UPR, which is a cytoprotective response to cope with ER stress, contributes to the survival of *S. cerevisiae* cells upon exposure to prodigiosin. However, this insight does not necessarily mean that prodigiosin damages *S. cerevisiae* cells mainly by inducing ER stress. We think that the same can be said for other ER stress stimuli such as ethanol. Whereas the UPR is thought to contribute to ethanol’s tolerance of *S. cerevisiae* cells [36], ethanol is likely to cause toxic effects on a wide range of biological molecules, such as cell membranes, in yeasts [37]. To comprehensively understand the effect of prodigiosin on *S. cerevisiae* cells, it would be intriguing to investigate the cellular response to prodigiosin using omics approaches in the future.

Some antibiotics, as well as prodigiosin, are known to act as ER stressors. For instance, tunicamycin, a secondary metabolite produced by *Streptomyces* species, inhibits the N-glycosylation of ER client proteins, leading to the induction of ER stress in a wide variety of eukaryotic species. From the viewpoint of selectivity of the toxic effect, it may be reasonable to assume that antibiotic chemicals produced by bacteria cause ER stress, which occurs only in eukaryotic species.

## 5. Conclusions

Through a study on *S. cerevisiae*, a widely used model organism, we demonstrated that prodigiosin provokes the UPR by inducing ER stress caused by the ER accumulation of unfolded proteins. Our study presents a new mode of action of prodigiosin, a prominent bacterial secondary metabolite. Nevertheless, it is still unclear what the primary target of prodigiosin is, which is a future research question.

## Figures and Tables

**Table 1:** Inhibition circle assay to assess sensitivity to prodigiosin.

Strain	Test Chemical (100 µL)	Diameter of Inhibition Zones	*p*-Value
*IRE1+*	DMSO only	Inhibition zone was not formed (no toxicity)	
*ire1∆*	DMSO only	Inhibition zone was not formed (no toxicity)	
*IRE1+*	Prodigiosin 1 mg/mL in DMSO	11.83 ± 0.07 mm	0.0019
*ire1∆*	Prodigiosin 1 mg/mL in DMSO	12.66 ± 0.14 mm	

SD agar plates on which overnight liquid cultures of the indicated strains had been spread were punched to make 8 mm diameter holes. After application of the test chemicals to the holes, the agar plates were incubated at 30 °C for 3 days. The diameter of inhibition zones was then measured. Data are presented as the average and standard deviation from dual biological replicates.

## Data Availability

Data are contained within the article and Supplementary Materials.

## Supplementary Materials

The following supporting information can be downloaded at: https://www.mdpi.com/article/10.3390/cimb46030116/s1, Figure S1: Chemical structure of prodigiosin; Figure S2: Integrity and purity of the prodigiosin sample; Figure S3: garose electrophoresis data to exhibit the HAC1 mRNA splicing upon treatment of cells with prodigiosin; Figure S4: Representative images of the inhibition circle.
