# Supplementary material for: Induction of Endoplasmic Reticulum Stress by Prodigiosin in Yeast Saccharomyces cerevisiae
Source: Curr Issues Mol Biol. 2024 Feb 26;46(3):1768–76. doi: 10.3390/cimb46030116 (PMC10969373; doi:10.3390/cimb46030116)

Figure S1

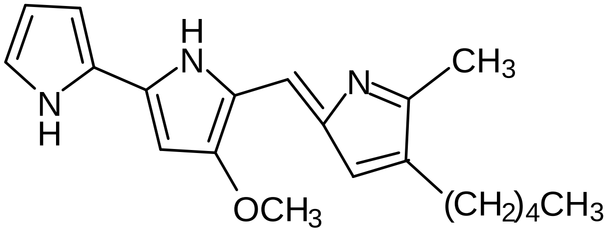


**Figure S1 Chemical structure of prodigiosin**

# Figure S2

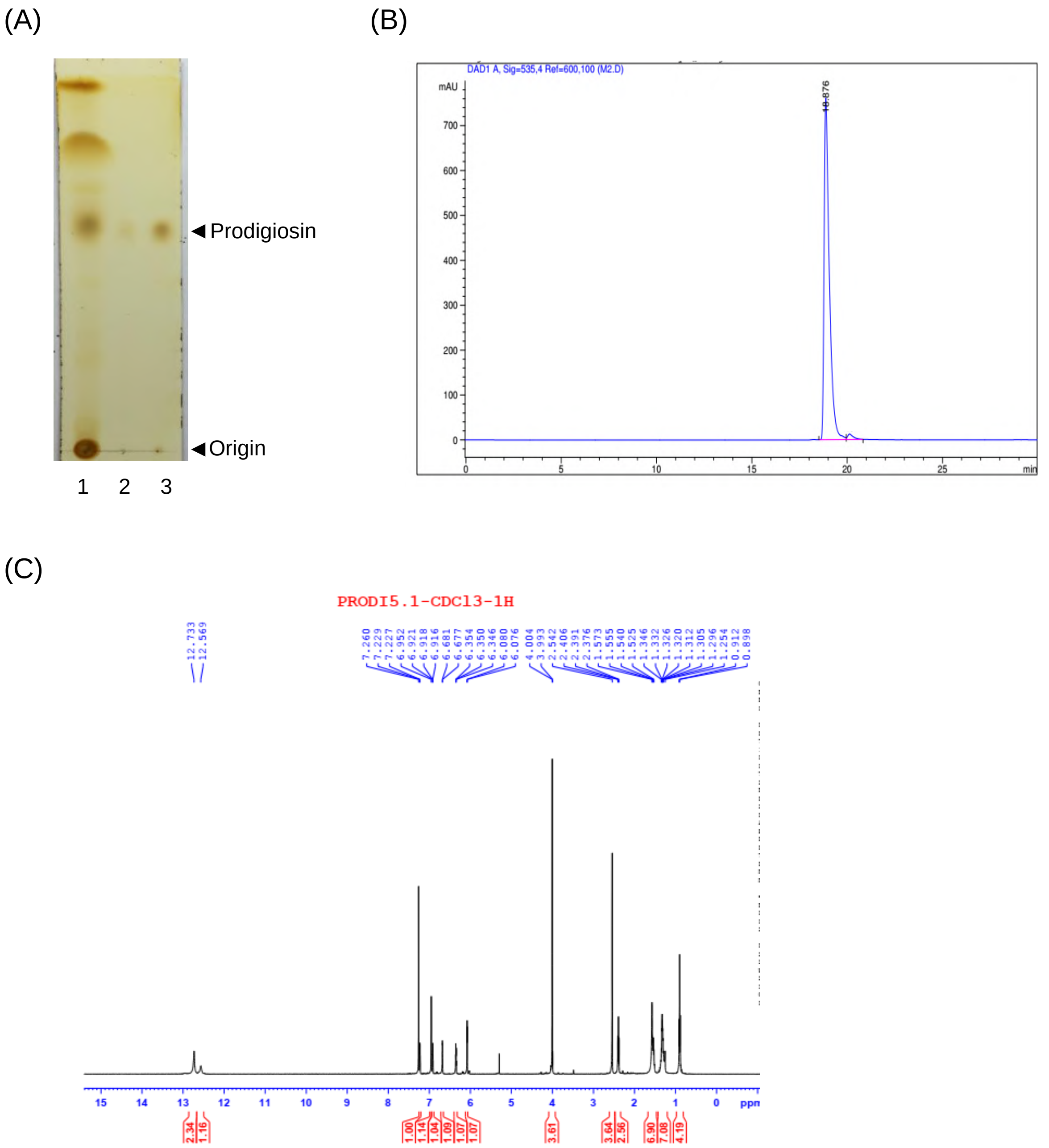


**Figure S2 Integrity and purity of the prodigiosin sample**

(A) TLC. Crude extract from S. marcescens (Lane 1), a commercial prodigiosin standard (Lane 2, Sigma-Aldrich), and our prodigiosin sample (Lane 3) were developed with the developing solvent of a hexane and ethyl acetate mixture (1:1) on a silica gel plate (0.25 mm thick silica gel 60 F254), which was then subjected to iodine staining. (B) HPLC. Our prodigiosin sample was analyzed by LC/MS 1100 Agilent Ion sources ESL (column: ODS C18, 3.0x150 mm, 3.5 μm; mobile phase: methanol:water=80:20). The molecular mass of the main peak (324.1) agreed with the theoretical molecular mass of prodigiosin. (C) $^{1}H$ NMR. Our prodigiosin sample was dissolved in $CDCl_3$ and analyzed by Bruker AVANCE-500MHz. The NMR spectrum matched that reported previously [19].

Figure S3

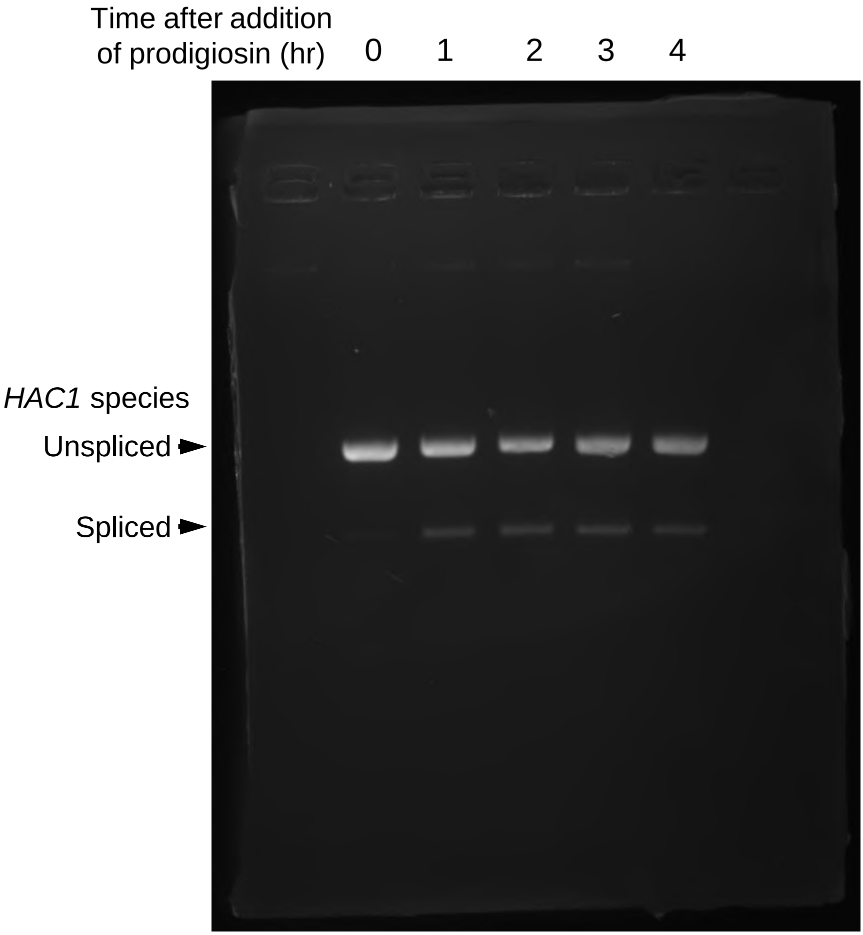


**Figure S3 Agarose electrophoresis data to exhibit the *HAC1* mRNA splicing upon treatment of cells with prodigiosin**

A representative sample set that had been analyzed by RT-qPCR in Fig. 1B were subjected to the conventional RT-PCR to amplify the *HAC1* species, and its products were fractionated by agarose gel electrophoresis. The ethidium-bromide fluorescent image of the gel are presented.

Figure S4

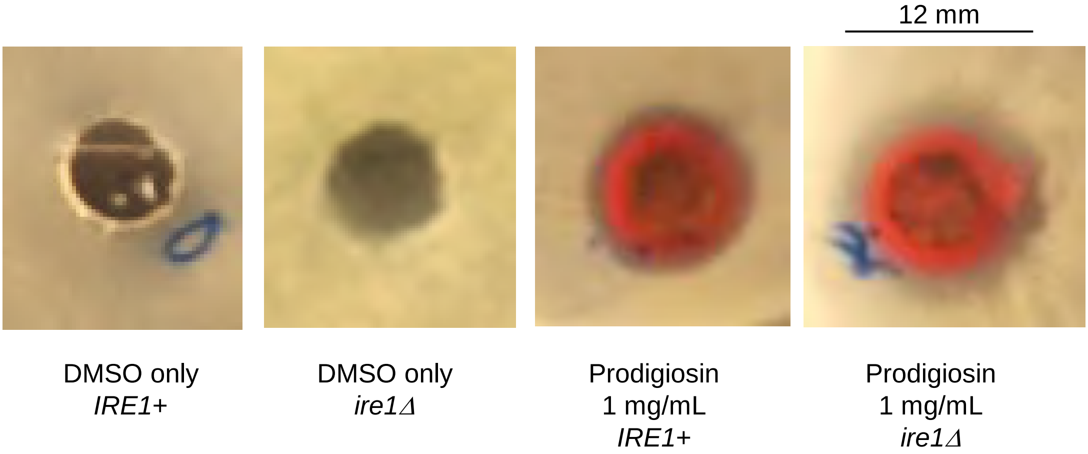


**Figure S4 Representative images of the inhibition circle**
Agar plate images taken in the experiment shown in Table 1 are presented.

An original image of Fig. S3

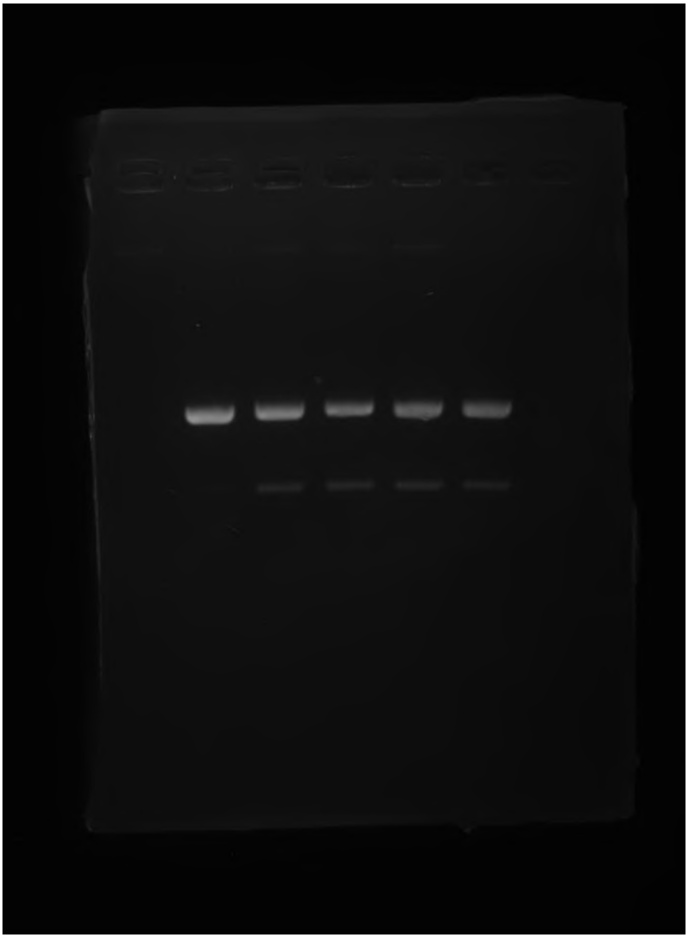

Supplement: Supplementary file 1 [file cimb-46-00116-s001.zip › cimb-2885638-supplementary.pdf]
